# Synthesis and characterization of amorphous precipitated silica from alkaline dissolution of olivine†

**DOI:** 10.1039/c8ra06257a

**Published:** 2018-09-21

**Authors:** Nadeem Raza, Waseem Raza, Silvia Madeddu, Henry Agbe, R. V. Kumar, Ki-Hyun Kim

**Affiliations:** Department of Material Science & Metallurgy, University of Cambridge UK; Govt. Emerson College, affiliated with Bahauddin Zakariya University Multan Pakistan; State Key Laboratory of Fine Chemicals, School of Chemical Engineering, Dalian University of Technology Dalian Liaoning 116024 China; Department of Civil and Environmental Engineering, Hanyang University Seoul 04763 Korea kkim61@hanyang.ac.kr

## Abstract

The high worldwide demand for amorphous precipitated silica (APS) materials, millions of tons worth billions of dollars, makes it worthwhile to further expand the techniques for synthesizing new, cheap, and environmentally friendly resources. In this research, amorphous precipitated silica was synthesized from alkaline dissolution of olivine using a mixture of NaOH and KOH and characterized; this combination resulted in better kinetics than those of the separate components. Experimental parameters (concentration of alkali, liquid/solid ratio, reaction time, and temperature) were optimized to provide maximum recovery of APS from olivine dissolution, which was then characterized using X-ray diffraction (XRD), scanning electron microscopy (SEM), transmission electron microscopy (TEM), Brunauer–Emmett–Teller (BET) N_2_ adsorption–desorption measurements, and thermogravimetric analysis (TGA). The APS possessed suitable morphology for use as an additive in polymers and in catalysis: a particle size below 10 nm, pore width of 5.59 nm, BJH adsorption cumulative pore volume of 0.96 cm^3^ g^−1^, BET surface area of 670.8 m^2^ g^−1^, and Langmuir surface area of 859.3 m^2^ g^−1^. The apparent activation energy of olivine dissolution with a mixture of NaOH/KOH was 43.6 kJ mol^−1^. The steps involved in creation of APS from olivine resulted in opportunities for carbon dioxide absorption, which could contribute to the production of valuable materials through decarbonation of exhaust gases.

## Abstract 2

The high worldwide demand for amorphous precipitated silica (APS) materials, millions of tons worth billions of dollars, makes it worthwhile to further expand the techniques for synthesizing new, cheap, and environmentally friendly resources.

## Graphical abstract

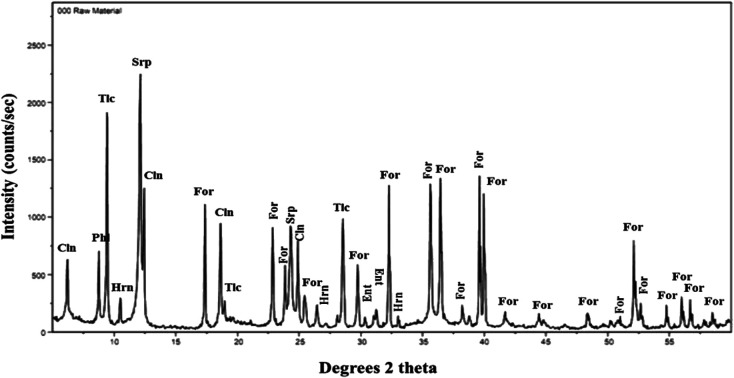

## 1. Introduction

Amorphous precipitated silica (APS) contributes to living standards in a wide range of applications, such as the reinforcement of elastomer products like tires, rubber goods, and drug delivery systems.^1,2^ APS is widely used in Europe for making energy efficient tires that have less friction, thus helping to reduce vehicle fuel consumption,^1^ and there is an increasing demand in North America and Asia^2^ for this application. Global industry analysis^3^ has reported that the worldwide demand for precipitated silica is expected to expand at a 5.5% compound annual growth rate between 2015 and 2023. According to this report, the global precipitated silica market was US$2.22 bn in 2015 and will increase to US$3.49 bn by 2023.^3^

To meet the increasing demand for quality amorphous precipitated silica, it is vital to find different silica precursors and cheaper production technologies. Two main technologies are used for the manufacture of APS: a pyrogenic method and a wet method.^4^ Pyrogenic APS powders for tire fillers are produced commercially by precipitation from sodium silicate, which is conventionally produced by high-temperature fusion of sodium carbonate and quartz sand.^1^ In the wet method, different lixiviants (*e.g.*, acids/bases and their salts) are used to extract silica in the form of either silicic acid or alkali metal silicate solutions, which are then neutralized to produce APS. This process does not require a high-temperature reaction and thus requires lower energy input than the pyrogenic route, which is considered less economically viable.^4^ The development of less energy intensive technologies for APS production would significantly contribute to the expansion of its industrial applications, especially as a reinforcing filler in the rubber and tire industry. Moreover, APS could have environmentally beneficial applications such as adsorbents or catalysts for selective removal and/or degradation of species contained in industrial effluents.^5^

Enormous effort has been exerted to find alternative raw materials for the production of APS by the wet process, such as rice husk, rice straw, bagasse ash, naturally occurring silicate rock (olivine), and photonic industrial wastes.^6–12^ However, the selection of silica raw material for commercial APS production has been governed by various factors such as its abundance, cost, availability, and the types and relative concentrations of impurities. Despite the abundance of rice husk, rice straw, and bagasse ash, their use as source materials for APS requires high energy consumption and additional purification steps for the removal of impurities. Similarly, the utilization of photonic waste cannot meet the high global demand for APS.

Magnesium silicate minerals have high potential for carbon dioxide sequestration through mineralization, which could offer an alternative to geological reservoirs for the storage of exhaust CO_2_.^13–15^ Geological surveys indicate the great global abundance of olivine ((Mg, Fe)_2_SiO_4_) and serpentine (Mg_3_Si_2_O_5_(OH)_4_),^16^ it has been estimated that these minerals are theoretically sufficient to fix the entire quantity of CO_2_ emitted globally by the combustion of fossil fuels.^17^ Moreover, as these minerals are rich in SiO_2_, they could potentially be used as a source of APS to provide a valuable by-product in carbon mitigation and to improve the economics of decarbonation. Coupling this process with CO_2_ sequestration would result in an innovative and environmentally benign technology for the synthesis of APS.

The chemical formula of olivine shows that it forms as a solid solution between it send products forsterite (Mg_2_SiO_4_) and fayalite (Fe_2_SiO_4_).^18^ Forsterite is normally preferred for CO_2_mineralization because it forms Mg carbonate minerals, which are stable and benign.^14^ Forsterite and serpentine usually contain iron impurities as well as sulfur, titanium, nickel, and chromium in smaller amounts. The production of APS from olivine can be achieved using acidic or alkaline solutions through the optimization of reaction conditions (*e.g.*, reaction temperature, concentration of acids or bases, and amount/particle size of olivine).^4,19^ For this purpose, comprehension of the reaction kinetics is critical as the dissolution of stable minerals is naturally a slow process. Nonetheless, relatively little is known about the influence of reaction parameters on the dissolution of silicate rocks and the subsequent synthesis of APS. The dissolution of forsteritic olivine has been carried out with HCl to reveal its dependence on pH and reaction temperature.^20^ This showed that the dissolution rate became more pH-dependent as the reaction temperature increased (at 65 °C and pH 5, the release of magnesium and silicon from forsterite was higher than that at pH 2). Similarly, Lieftink and Geus^10^ used sulfuric acid as a leaching agent to investigate the effect of olivine particle size on the porosity and surface area of nano-silica. They obtained silica with very small particle sizes ranging from 8 to 25 nm. In this study, the grain size of olivine was addressed, but no attention was given to other kinetic reaction parameters (reaction temperature, rate controlling steps, or stoichiometry of reactants) that affect the dissolution rate of olivine and the resulting textural properties of APS.

Lazaro *et al.*^4^ also used sulfuric acid as a lixiviant for the dissolution of olivine to obtain 95% pure APS and assessed the characteristics of synthesized nano-silica. They concluded that the olivine dissolution process in a dilute acidic solution could be a convenient alternative to the traditional pyrogenic methods used for the bulk production of nano-silica because it is less energy intensive. However, no attention was paid to deducing the rate-determining step in the acid dissolution of olivine. Moreover, studies concerning the dissolution of olivine in acidic media necessarily require additional steps for purification.^4,10,21,22^ Despite the fact that mineral acids are good lixiviants, they exhibit a greater tendency to dissolve impurities such as iron from the minerals along with the components of interest (magnesium and silicon), leading to contamination of the products.^23^ On the other hand, alkaline solutions cannot dissolve iron from minerals because of their weak dissolving power. Therefore, the use of alkaline extracting agents is advantageous compared to that of acidic solvents. Most studies carried out using both wet and pyrogenic silica synthesis approaches report that the surface area of APS is in the range of 100–300 m^2^ g^−1^, while most catalytic and sorption processes generally demand higher surface area. Further studies are greatly needed to produce APS from easily available and abundantly occurring raw materials using economically viable extraction technologies. Moreover, an increase in the surface area of the APS would significantly expand its market for certain applications.

In this study, the alkaline dissolution of olivine is carried out using NaOH and KOH to synthesize APS with increased surface area. The study aims to maximize the synthesis efficiency of APS by focusing on the alkaline extraction efficiency through an evaluation of the effects of various experimental parameters, including concentration of alkalis, liquid to solid ratio, reaction time, and temperature, on the conversion and morphology (*i.e.*, surface area)of APS particles. The determination of optimal reaction conditions through reaction kinetics and rate limiting steps is vital for the large scale commercial production of various chemicals. Therefore, the kinetics of olivine dissolution in alkaline media are also analyzed to determine the rate controlling step and to optimize the reaction conditions.

## 2. Experimental

### 2.1 Sample preparation and analysis

The raw material olivine, mined from ultramafic rock formations in Norway, was supplied by Sibelco. A number of representative samples were collected from the raw material, crushed by a jaw crusher, ground by ball milling, screened by Tyler standard sieves to obtain different size distributions, and dried in an oven at 100 °C for 24 h. Chemical composition of olivine was determined by a third party, and analytical results are shown in Table S1.†

**Fig. 1:**
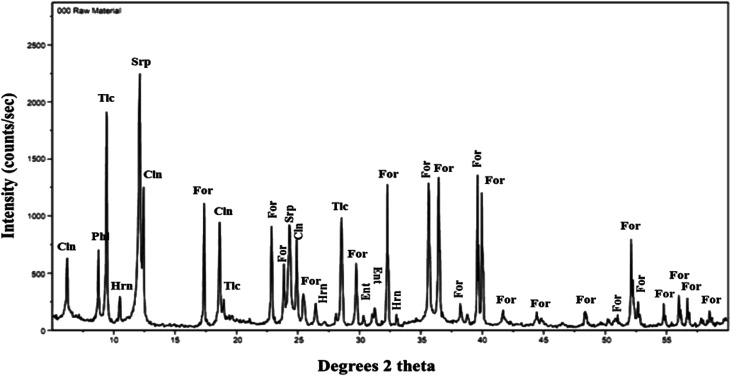
XRD pattern of raw material olivine, with assigned diffraction peaks: For: forsterite, Hrn: hornblende, Srp: serpentine, Cln: clinochore, Tlc: talc, Ent: enstatite, and Phl: phlogopite.

The mineralogical composition of olivine was determined by X-ray diffraction (XRD) using a D8 Bruker diffractometer with Cu Kα radiation source of wavelength *λ* = 1.54 Å, 2*θ* scans of 3 to 80°, and a step size of 0.029° 2*θ*. The software High Score Plus was used to analyse the diffraction pattern and identify the major and minor phases present. The diffraction pattern of olivine is shown in Fig. 1, indicating that the material mainly consisted of forsterite, along with other minor magnesium-bearing minerals: hornblende, serpentine, clinochore, talc, enstatite, and phlogopite.

Scanning electron microscopy (SEM) (Jeol 5800LV) was used to image the morphology of the olivine sample. Energy dispersive X-ray (EDX) was used to confirm the elemental composition in selected spots. The SEM image in Fig. S1a† shows that the olivine particles had a wide size distribution with an average size below 70 μm and certain regions of bigger particles. These observations are in agreement with the analysis of olivine previously conducted by Madeddu *et al.*, who measured a distribution of particle size with an average around 25 μm, with 90% of the particles <58 μm.^14^ The EDX spectrum of the olivine sample (Fig. S1b†) indicates the presence of Mg, Si, Fe, and O as major elements.

### 2.2 Experimental procedure

Ten grams of olivine with particle size between 74 and 125 μm were added to a polytetrafluoroethylene (PTFE) reactor vessel containing a fixed concentration of the alkaline solution used to dissolve the olivine. The solution was prepared by mixing known weights of powdered NaOH and KOH with a fixed amount of deionized water to obtain a dense slurry. The reactor was closed with an inverted lid to avoid frequent escape of vapours and heated to a fixed reaction temperature and time in an electric furnace (Elite BCF 13/5-2408CP). The chemical reaction that occurred can be written as follows:

1 Mg_2_SiO_4(s)_ + 2NaOH_(s)_ + H_2_O → 2Mg(OH)_2(↓)_ + Na_2_SiO_3(aq)_

After completion of the reaction, 50 mL deionized water was mixed with the solid product, and the solution was stirred for 30 minutes to extract the soluble species. Afterward, the solid products were separated by vacuum filtration using Whatman filter paper of grade 6. The filtrate solution was then reacted with a dilute solution of hydrochloric acid (0.1 M HCl) to precipitate amorphous silica while keeping the pH of the solution around 2. The precipitated brucite from reaction (1) is a very effective decarbonation mineral, as shown in separate studies by this group^14^ and others.^24^ The reaction that yields APS can be written as follows:

2 Na_2_SiO_3(aq)_ + 2HCl_(aq)_ → 2NaCl_(aq)_ + 2H_2_O + SiO_2(↓)_

It has been reported that the colloidal solution of silica obtained from olivine dissolution is unstable in the pH range of 0.5–1, and that silica particles start to agglomerate and flocculate with an increase in particle size.^25,26^ The rate of agglomeration of APS is mainly affected by the pH of the solution and is catalysed by a higher concentration of hydrogen ions.^10,23^ APS was filtered from the liquor solution, washed with deionized water to remove the settled impurities, dried in an oven for 5 h, and stored in glass vials for further analysis. The overall process for the production of APS by alkaline leaching of olivine is shown in Fig. S2.†

The percent recovery of APS from olivine was calculated using the following equation:3
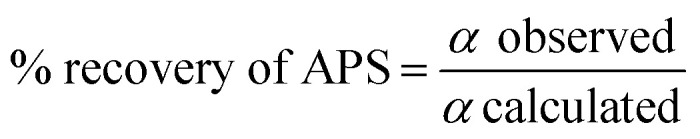
Here, *α* observed is the fraction of APS obtained experimentally from the dissolution of olivine, while *α* calculated indicates the theoretical concentration of silica contained in given amount of olivine based on the chemical analysis shown in Table S1.† In carbonation studies of serpentinite rocks, Kumar *et al.* reported that HCl can be replaced with CO_2(aq)_ in order to precipitate APS while extracting CO_2(g)_ in the form of soluble sodium carbonate or bicarbonate, which are also valuable by-products.^27,28^ Thus, both reactions (1) and (2) offer opportunities for decarbonation while producing important chemicals/materials to significantly offset the cost of decarbonation. In order to make further advances in carbon removal from exhaust gases in industrial activities, this work focused on the leaching and precipitation reactions as the most important steps for optimization.

## 3. Results and discussion

### 3.1 Optimization of reaction parameters

#### 3.1.1 Effect of reaction temperature on the leaching of silica from olivine

**Fig. 2:**
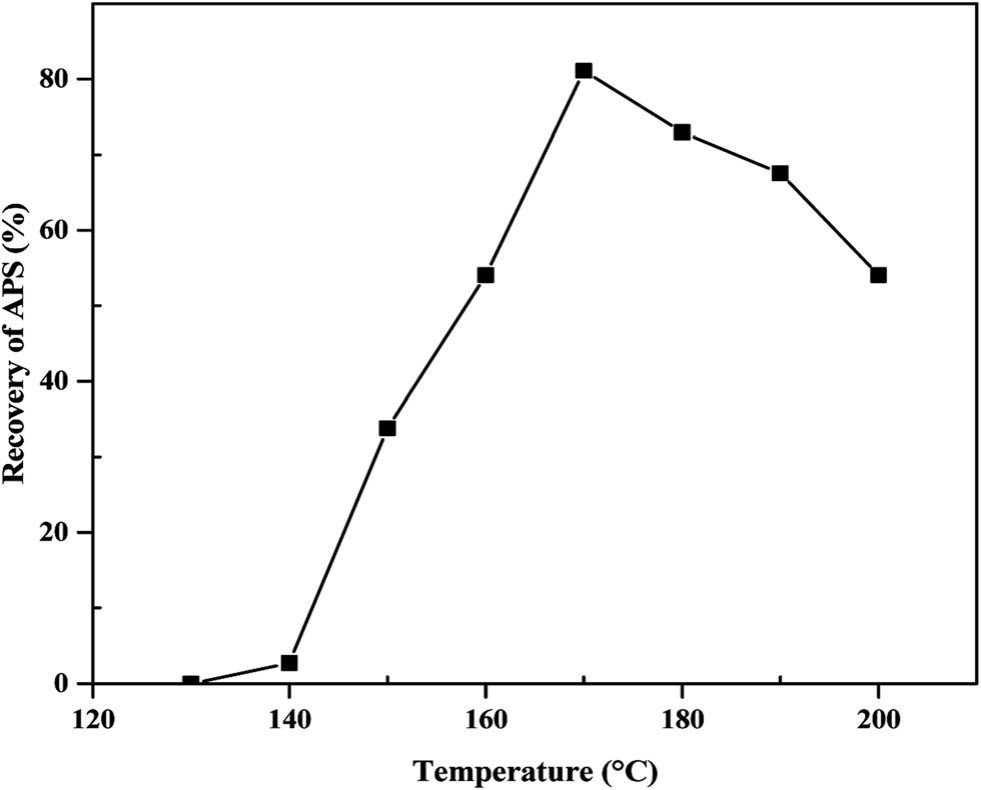
Effect of reaction temperature on percent recovery of APS from olivine by leaching (mass of bases 14 g, H_2_O content 0.8 mL, and reaction time 5 h for 10 g olivine).

A set of experiments was carried out to determine the effect of reaction temperature on the leaching kinetics of olivine by varying the temperature from 130 to 200 °C. The results are shown in Fig. 2 and demonstrate that the recovery of APS from olivine was most effective at a temperature of 170 °C. Reaction temperatures below 170 °C were not able to break the textural structure of olivine and thus led to poor recovery of APS. On the other hand, above 170 °C, rapid dehydration of the reaction mixture hindered the progress of the reaction.

#### 3.1.2 Effects of alkali concentration and liquid to solid ratio on the recovery of APS from olivine

A series of experiments was carried out to determine the influence of NaOH on the dissolution kinetics of olivine. However, it was observed that the leaching of olivine did not occur effectively in the presence of NaOH alone. Therefore, an experiment was devised to study the combined behaviour of NaOH and KOH, in which the addition of KOH with equal weight proportion of each alkali resulted in a significant positive effect, as described below. The concentration of each of the two alkalis varied from 5 to 9 g per 10 g of olivine in order to study their combined effect (at 50% of each) on the leaching of olivine. As shown in Fig. S3,† the highest APS recovery was obtained from olivine when 7 g NaOH + 7 g KOH reacted with 10 g olivine, which is higher than the stoichiometric amount for the reaction given in eqn (1). This observation indicates that a highly concentrated alkaline solution is required to digest the olivine and to extract silica.

The volume of water in the reaction mixture also varied from 0.5 to 1.2 mL per 10 g of olivine. Fig. S4† illustrates that, within the range of water amount tested in this study, there was an optimum value for APS extraction, corresponding to 0.8 mL per 10 g of olivine. When the amount of water was higher or lower than 0.8 mL, a negative impact on olivine leaching was observed, as shown in Fig. S4.† At concentrations below 0.8 mL, the liquid content may not have been sufficient to favour olivine dissolution, while at higher concentrations, it is possible that the effects of alkali dilution reduced the leaching of APS.

#### 3.1.3 Effect of reaction time

A series of experiments was performed to determine the effect of reaction time on the recovery of APS from olivine. Fig. S5† shows that the maximum conversion of olivine to APS was 86.8%, achieved at 6 h reaction time. Further increase in reaction time did not result in any increase in percent yield of APS. This may be attributable to the observation made during the experimental work that, with an increase in reaction time, the products became very hard, making it impossible to extract silica completely.

### 3.2 Characterization of APS

**Fig. 3:**
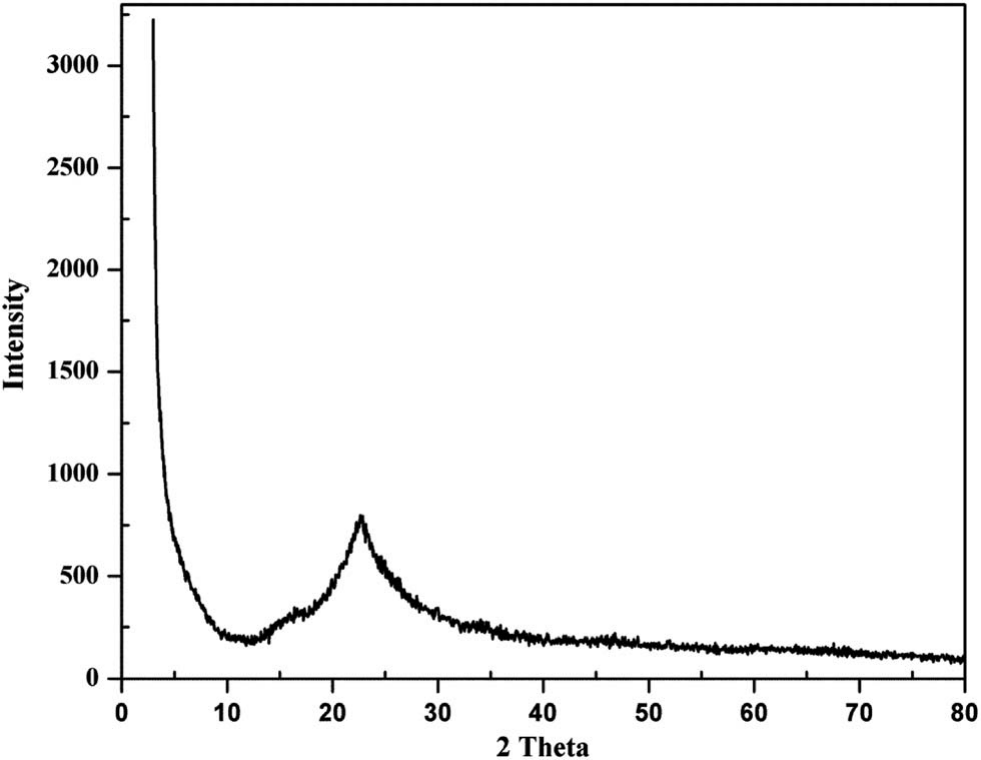
XRD pattern of amorphous precipitated silica.

**Fig. 4:**
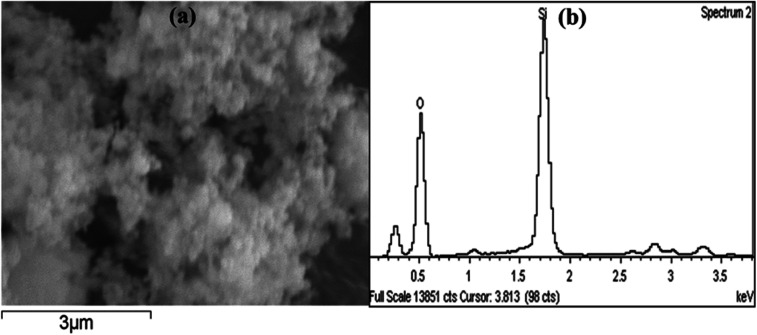
(a) SEM micrograph of APS powder. (b) EDX pattern of APS powder.

Amorphous precipitated silica obtained from alkaline dissolution of olivine was characterized by XRD, SEM, transmission electron microscopy (TEM), gas adsorption analysis, and thermogravimetric analysis (TGA). To analyse the APS, the same X-ray diffractometer and diffraction parameters were used for consistency. The XRD pattern of APS shown in Fig. 3 shows that the APS sample lacked any crystalline phase and exhibited an amorphous nature. The morphology of the APS was studied used SEM (Jeol-5800LV). Fig. 4a shows the fluffy and porous nature of APS, and that the most of the APS particles were smaller than 1 μm. The EDX spectrum of APS (Fig. 4b) shows that the APS sample was devoid of any inclusions or impurities.

**Fig. 5:**
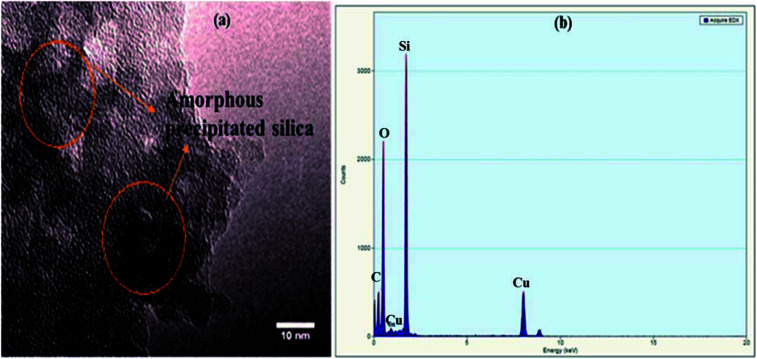
(a) TEM image of APS powder. (b) TEM-EDX spectrum of APS powder.

TEM (FEI Tecnai G2 F20) operating at an acceleration potential of 200 kV was used to find the particle size distribution of the APS sample. A TEM image and TEM-EDX spectrum are shown in Fig. 5a and b, respectively. Fig. 5a illustrates the absence of any crystal planes in the synthesized APS, while most particles were below 10 nm. In Fig. 5b, the peaks for Cu and C are from the sample holder and substrate, respectively, while the presence of silicon and oxygen confirms the high purity of the synthesized APS.

**Fig. 6:**
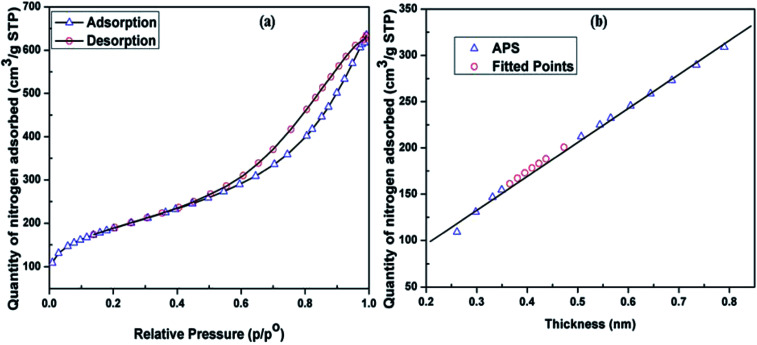
(a) Isotherm linear plot of synthesized APS. (b) *t*-plot showing the physisorption of APS.

A gas physisorption study of the APS sample was carried out using a Micromeritics TriStar 3000. The APS sample was soaked for 12 h at 150 °C and analysed using nitrogen gas. The relative physisorption curves in Fig. 6a and b show high rates of sorption and desorption of nitrogen in the APS sample. Brunauer–Emmett–Teller (BET) surface area, Langmuir surface area, and micropore surface area were determined using BET, *t*-plot, and Barrett–Joyner–Halenda (BJH) methods, respectively.^29–31^ The results showed BET and Langmuir surface are as of 670.8 and 859.32 m^2^ g^−1^, respectively, demonstrating that the APS produced from olivine in this work had a high surface area. The average adsorption pore width of APS was found to be 5.59 nm with 0.955 cm^3^ g^−1^ BJH cumulative adsorption pore volume, indicating that the APS synthesized by alkaline dissolution of olivine had a nanoporous nature.

The thermal stability of APS was determined by TGA using a heating rate of 10 °C min^−1^. The TGA curve in Fig. S6† indicates two regions of weight loss, a lower temperature weight loss due to evaporation of weakly bonded (physisorbed) water molecules and a higher temperature loss during condensation of silanol groups.^4^ The overall weight loss of the APS sample was 11%; 8% weight loss occurred below 200 °C, while the remaining 3% took place between 200–500 °C.

The characterization of APS using different instrumental techniques confirms that the amorphous precipitated silica obtained from the alkaline leaching of olivine exhibited high surface area (670.8 m^2^ g^−1^) and very small particle size (below 10 nm). APS produced by this method could be employed in various applications, especially catalysis, because of its porous surface.

A summary of the BET surface area, particle size, pore size, and percent yield of nano-sized silica synthesized from different raw materials using various extractants reported in the literature is given in Table 1. Silica obtained from other studies may possess high BET surface area and low particle size, but the APS produced in this study has the best combination of small particle size, high surface area, small pore size, and high porosity. These properties are ideal for the application of APS as a carrier and support for catalysts such as silica-supported chromium, copper, aluminum phosphate, and nickel.

**Table 1:** Comparison of physical characteristics of silica obtained from different raw materials

Serial #	Raw material	BET surface area (m^2^ g^−1^)	Particle size (nm)	Pore size (nm)	Yield (%)	Reagent/method used	Reference
1	Olivine	100–300	10–25	>10	66–83	H_2_SO_4_	4
2	Rice hull ash			50–60	94.6	NH_4_F	6
3	Rice straw	509.5	Nano discs	5.8	90.8	NaOH	7
4	Rice husk and bagasse	233, 271	46, 48			NaOH	8
5	Olivine	100–400	8–25	>10		H_2_SO_4_	10
6	Photonic waste	585	Spherical particles	9.1		Salt templated aerosol spray	12
7	Olivine	670.8	<10	5.59	86.8	NaOH and KOH	This study

### 3.3 Kinetic analysis

In solid–fluid systems, the reaction rate is governed by one of the following kinetic mechanisms: diffusion through fluid film, diffusion through ash or product layer, or surface-controlled chemical reaction.^32–34^ In a general form, the solid–fluid reaction can be represented as

4 A_(fluid)_ + B_(solid)_ → C_(products)_

In the absence of ash or product layer formation, only two controlling mechanisms (surface chemical reaction and diffusion through fluid film) are important in governing the leaching kinetics of olivine.^23,33–36^ The rate equations for surface chemical reaction and diffusion through fluid film can be represented by eqn (5) and (6), respectively:

5 1−(1 − *α*)^1/3^ = *k*_S_*t*

6 *α* = *k*_D_*t*

Here it is assumed that *α* is the fraction of olivine dissolved, and *t* is the reaction time (min), and *k*_S_ and *k*_D_ are the reaction rate constants for surface chemical reaction and diffusion through fluid film, respectively. In order to determine the rate-limiting step for the leaching of silica from olivine, the experimental data were analysed using statistical and graphical approaches. From an analysis of correlation coefficients, it was determined that the leaching of olivine in alkaline media follows a surface chemical reaction-controlled mechanism. The value of the rate constant *k*_S_ was evaluated by plotting 1−(1 − *α*)^1/3^*vs. t*, as shown in Fig. S7.†

Using the Arrhenius equation, the rate constant can be expressed as

7 *k*_S_ = *A*_o_e^−*E*_a_/*RT*^

Here, *A*_o_ is the Arrhenius constant, *E*_a_ the energy of activation, *R* the ideal gas constant, and *T* the reaction temperature. By comparing eqn (5) and (7), the integral rate expression can be written as

8 1−(1 − *α*)^1/3^ = *A*_o_e^−*E*_a_/*RT*^*t*

In order to determine the values of the activation energy and Arrhenius constant, ln *k*_S_ and (1/*T*) values were plotted, as shown in Fig. S8.†

Inserting the values for the activation energy and Arrhenius constant, eqn (8) can be written as9
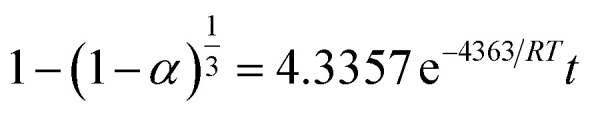


The value of the activation energy (43.6 kJ mol^−1^) in eqn (9) for the alkaline leaching of olivine is in agreement with values obtained in previous studies of fluid–solid reaction systems.^23,37–40^ For example, Abdel-Aal^37^ described that, for a chemically controlled process, the value of the activation energy is usually greater than 41.84 kJ mol^−1^. In most previous studies that have explored cheap and viable techniques to meet growing demand of APS, extraction of APS was achieved by acidic dissolution of olivine rocks. In those studies, there was not sufficient effort made to optimize the parameters that control the reaction kinetics or produce APS with high surface area (generally required for catalysis and drug delivery systems). Moreover, this approach (acid leaching) requires additional purification steps to remove the iron contained in olivine rocks. Optimization of reaction parameters is also generally required for large-scale commercial production. In this research, the reaction kinetics and relevant reaction parameters for the leaching of olivine in basic media were optimized to obtain APS with increased surface area. Specifically, the alkaline leaching of olivine was optimized with the following reaction conditions: mass of olivine: 10 g; mass of alkalis: 14 g; temperature: 170 °C; volume of water: 0.8 mL; reaction time: 5 h. Based on this newly proposed approach, we were able to synthesize high purity, high surface area APS appropriate for applications in various fields, especially catalysis.

## 4. Conclusions

A large amount of amorphous precipitated silica with high surface area is required for many applications in the automotive, food, pharmaceutical (drug delivery systems), and chemical industries. In order to meet the increasing world demand for APS, cheaper raw materials and more economical extraction strategies are essential. To this end, the present study describes an innovative method to synthesize APS with high surface area from a highly abundant and cheap mineral source of silica (olivine) using a relatively low-cost and less energy intensive process. Amorphous precipitated silica was successfully extracted from naturally occurring olivine reacted with a mixture of NaOH, KOH, and H_2_O, and the product demonstrated the chemical and physical properties required for industrial applications (surface area of 670.8 m^2^ g^−1^, particle size below 10 nm, and high thermal stability). It was found that the kinetics of olivine dissolution in alkaline media followed the surface chemical reaction model with an activation energy of 43.6 kJ mol^−1^. Moreover, it was successfully demonstrated that, for the synthesis of APS from olivine, this method is an alternative process for producing APS while simultaneously providing an opportunity for decarbonation of exhaust gases from industrial processes. The availability of many processes such as the one described here can cumulatively impact the rapid deployment of decarbonation technologies. This technique will not only provide a way to mitigate the substantial levels of CO_2_ released by industry, but could also help in the production of industrially viable chemicals.

## Conflicts of interest

The authors declare that there is no conflict of interest in this work.

## Supplementary Material

RA-008-C8RA06257A-s001
