# Correction: Synthesis and characterization of amorphous precipitated silica from alkaline dissolution of olivine

**DOI:** 10.1039/c8ra90080a

**Published:** 2018-10-25

**Authors:** Nadeem Raza, Waseem Raza, Silvia Madeddu, Henry Agbe, R. V. Kumar, Ki-Hyun Kim

**Affiliations:** Department of Material Science & Metallurgy, University of Cambridge UK; Govt. Emerson College, Bahauddin Zakariya University Multan Pakistan; State Key Laboratory of Fine Chemicals, School of Chemical Engineering, Dalian University of Technology Dalian Liaoning 116024 China; Department of Civil and Environmental Engineering, Hanyang University Seoul 04763 Korea kkim61@hanyang.ac.kr

## Abstract

Correction for ‘Synthesis and characterization of amorphous precipitated silica from alkaline dissolution of olivine’ by Nadeem Raza *et al.*, *RSC Adv.*, 2018, **8**, 32651–32658.

The authors regret that there were some errors in the synopsis of ref. 4 (A. Lazaro, H. Brouwers, G. Quercia and J. Geus, *Chem. Eng. J.*, 2012, **211**, 112–121) in the original article. There was a misleading comment in the second last paragraph of section 1 (Introduction) of the article. Readers should disregard the following sentence:

“However, no attention was paid to deducing the rate-determining step in the acid dissolution of olivine.”

This sentence is not appropriate as Dr Lazaro has already published an article in 2015, shown as ref. 1 below, which described the rate determining step in the acid dissolution of olivine. This reference should have been cited at this point in the introduction in the original article.

If the authors do not draw attention to the aforementioned sentence, it may mislead readers regarding the presence of previous efforts made in this subject. They thank Dr Lazaro, the lead author of ref. 4, for bringing this to their attention.

The Royal Society of Chemistry apologises for these errors and any consequent inconvenience to authors and readers.

## Supplementary Material
