# Supplementary material for: Synthesis and characterization of amorphous precipitated silica from alkaline dissolution of olivine
Source: RSC Adv. 2018 Sep 21;8(57):32651–8. doi: 10.1039/c8ra06257a (PMC9086262; doi:10.1039/c8ra06257a)
Supplement: RA-008-C8RA06257A-s001 [file RA-008-C8RA06257A-s001.pdf]

Electronic Supplementary Material (ESI) for RSC Advances.
This journal is © The Royal Society of Chemistry 2018

Supplementary Material

Synthesis and characterization of amorphous precipitated silica from alkaline dissolution of olivine

Nadeem Raza,[a,b] Waseem Raza,[c] Silvia Madeddu,[a] Henry Agbe,[a] R.V. Kumar,[a] Ki-Hyun Kim*[d]

[a]*Department of Material Science & Metallurgy, University of Cambridge United Kingdom;*[b]*Govt. Emerson College, affiliated with Bahauddin Zakariya University, Multan;* [c]*State Key Laboratory of Fine Chemicals, School of Chemical Engineering, Dalian university of Technology, Dalian, Liaoning, 116024 China;* *[d]*Department of Civil and Environmental Engineering, Hanyang University, Seoul 04763, Korea*

Table S1. Chemical analysis of olivine

Serial #	Component	[Wt.%]
1	MgO	40.0
2	Fe_2O_3	9.5
3	Al_2O_3	0.7
4	CaO	0.4
5	SiO_2	39.0
6	Loss on ignition	11.4 %

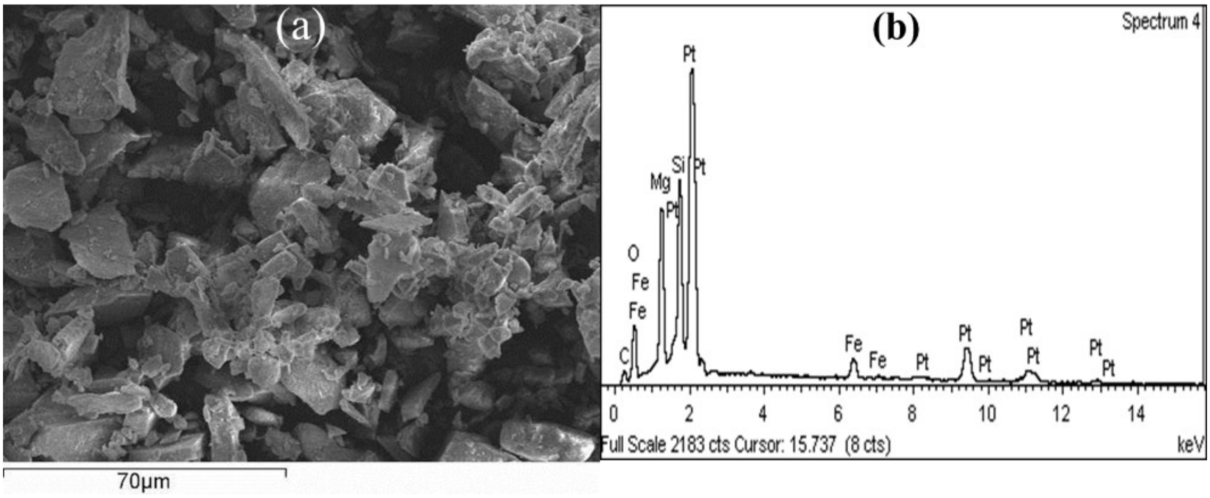

Fig. S1 Morphological characteristics of ground olivine(a)SEM image (b) EDX spectrum

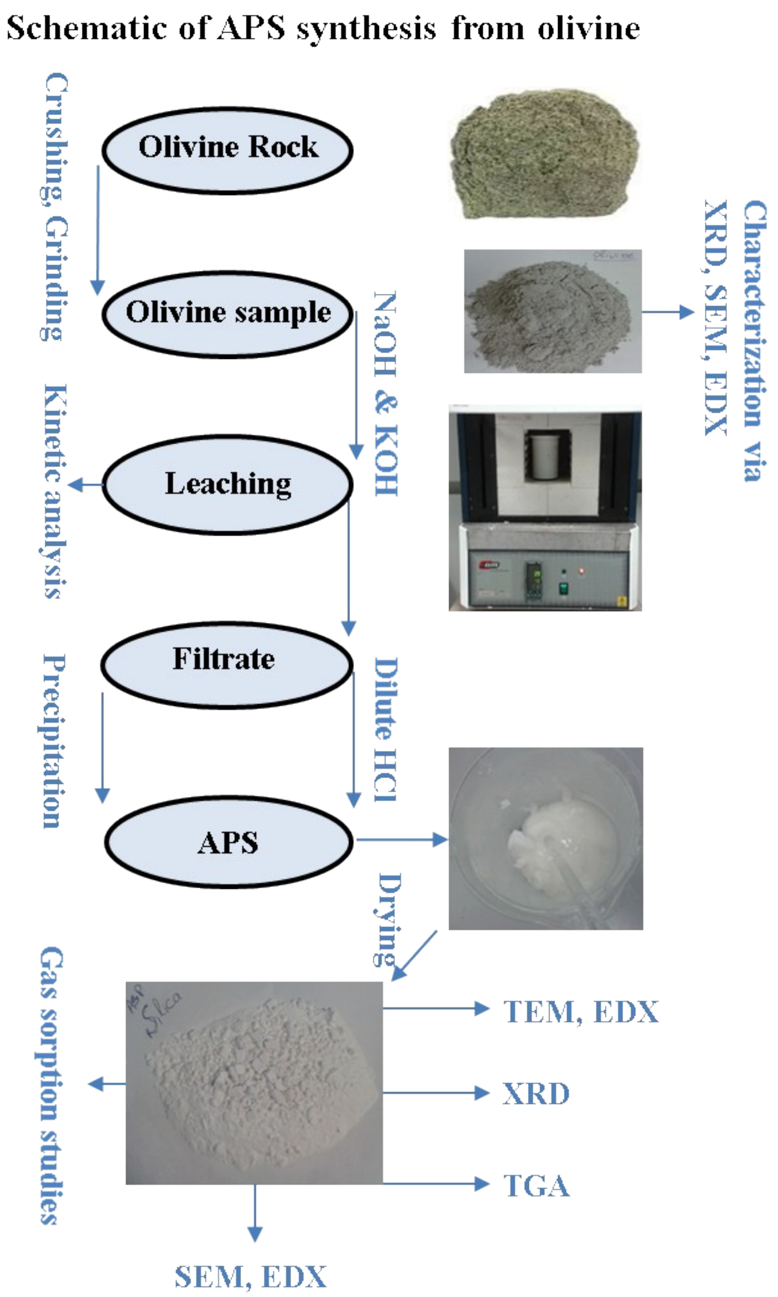

Fig. S2 Schematic of APS synthesis from olivine using alkaline dissolution.

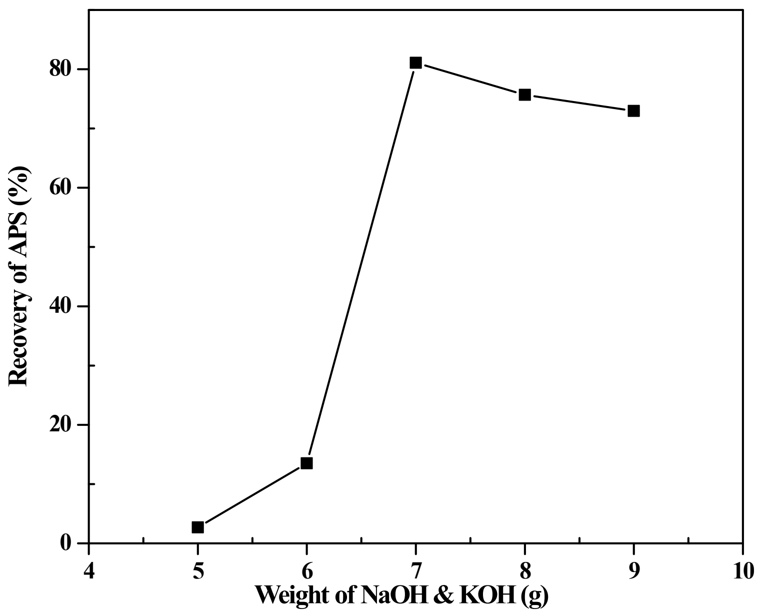

Fig. S3 Effect of concentration of NaOH and KOH on the leaching of silica from olivine (Temperature 170°C, H_2O content 0.8 mL, and reaction time 5 h for 10 g olivine).

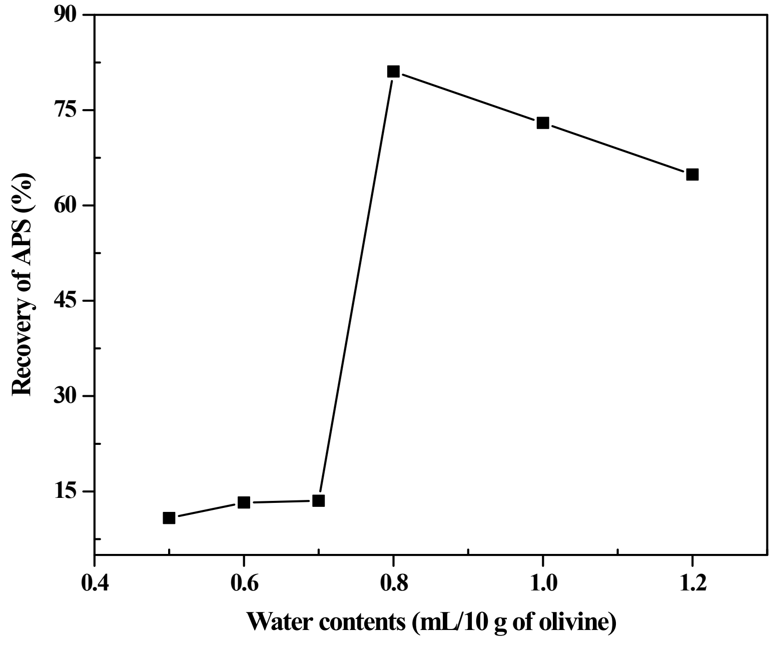

Fig. S4 Effect of water content on the leaching of silica from olivine (Temperature 170 ^{o}C, mass of bases 14 g, reaction time 5 h for 10 g olivine).

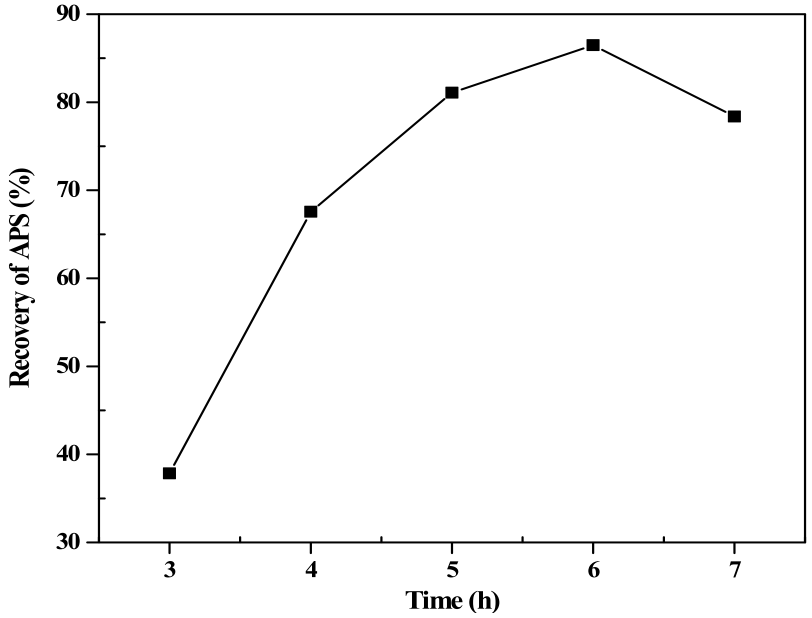

Fig. S5 Effect of reaction time on leaching of APS from olivine (Temperature 170 ^{o}C, H_2O content 0.8 mL, mass of bases 14 g for 10 g olivine).

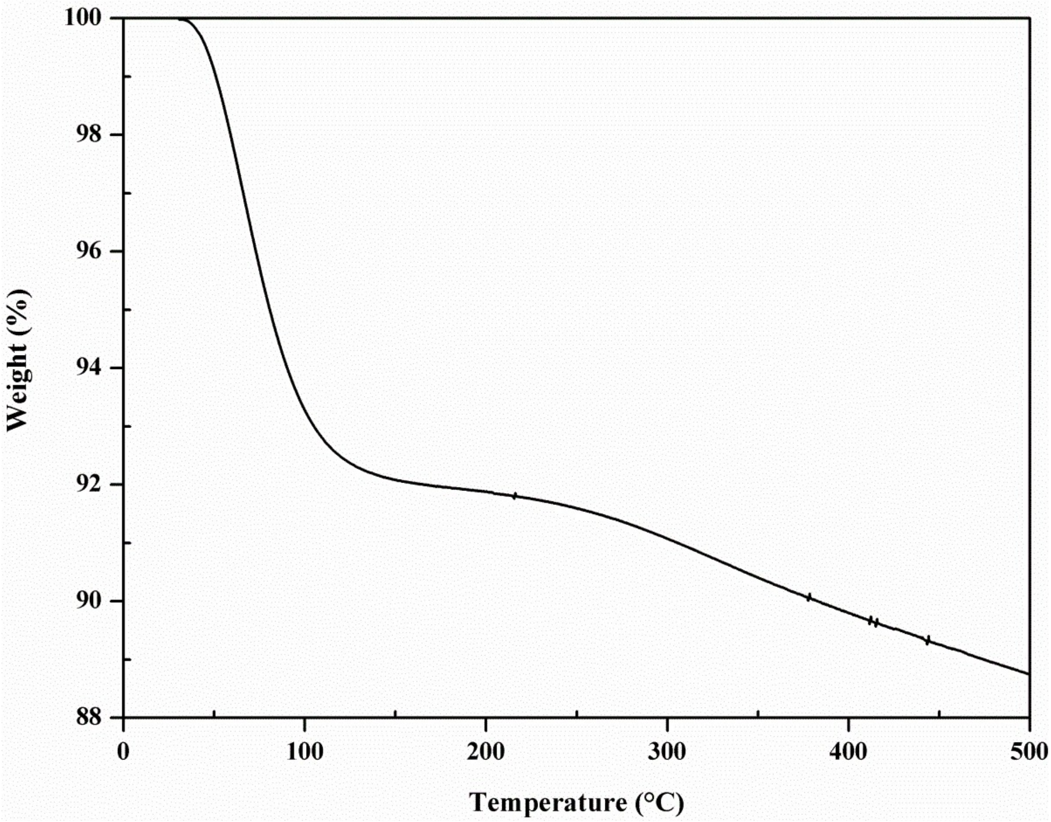

Fig. S6 TGA curve of APS.

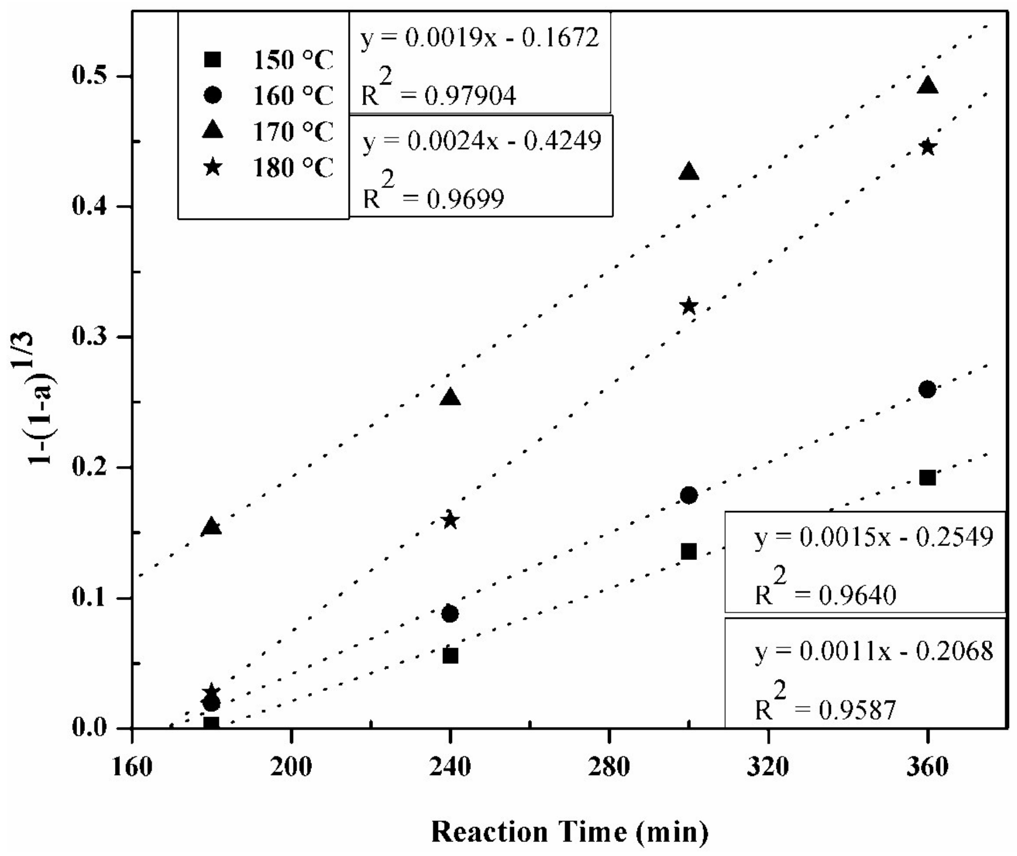

Fig. S7A plot of$1-(1-\alpha)^{1/3}$vs.reaction time.

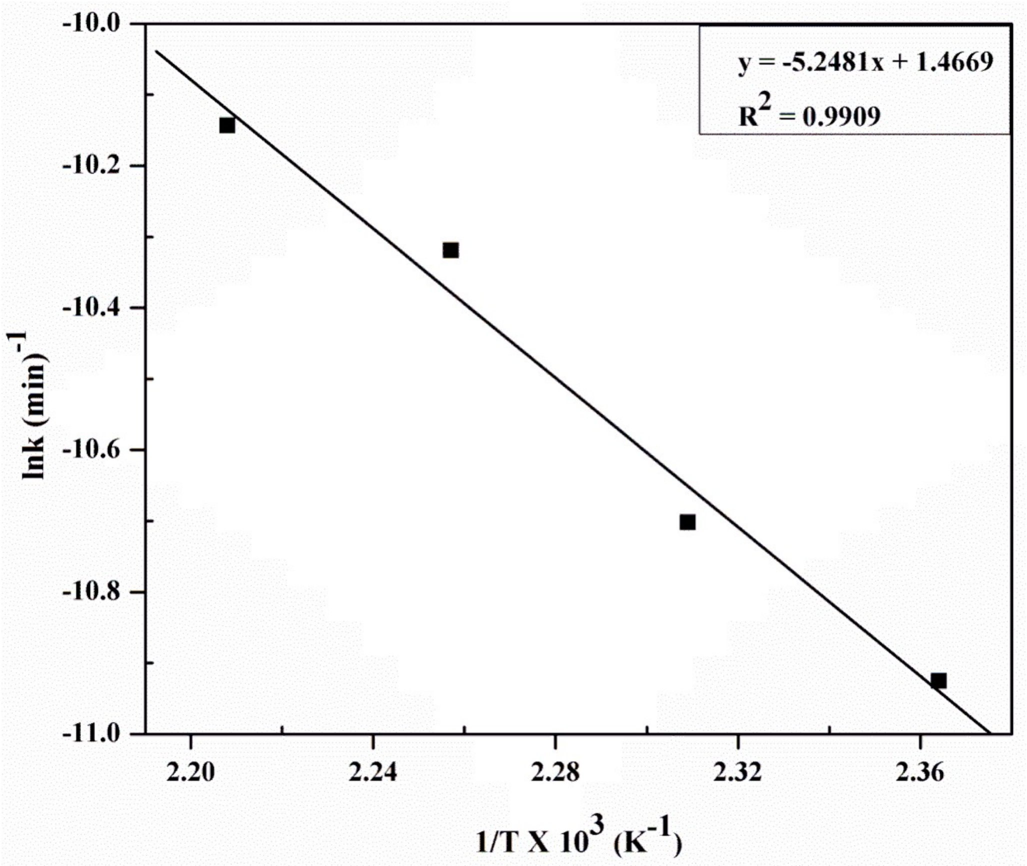

Fig. S8 Arrhenius plot.
